# Job Satisfaction and Associated Factors Among Primary Healthcare Workers: A Cross-Sectional Study From Qassim Region, Saudi Arabia

**DOI:** 10.7759/cureus.62969

**Published:** 2024-06-23

**Authors:** Norah H Alhaqqas, Amel A Sulaiman

**Affiliations:** 1 Family Medicine, Family Medicine Academy, Qassim Health Cluster, Buraidah, SAU

**Keywords:** nurses, physicians, primary health care, qassim, healthcare, job satisfaction

## Abstract

Background

The healthcare sector is one of the most important sectors in any country. Any disruption in the productivity of the workforce majorly affects healthcare entities. Job satisfaction directly interferes with the individual’s productivity. Hence, the job satisfaction of healthcare workers (HCWs) is a fundamental issue to discuss, investigate, and study to improve the outputs to their maximal levels, especially if limited studies are done in this regard.

Methodology

A descriptive, cross-sectional, facility-based study was conducted among 302 HCWs in 30 primary healthcare centers (PHCCs) in the Qassim region, Saudi Arabia, using a pretested, validated, electronically self-administered “Satisfaction of Employees in Health Care” (SEHC) questionnaire. Our sample included physicians, nurses, pharmacists, dental workers, and lab and radiology technicians. Data were analyzed using SPSS version 29 (IBM Corp., Armonk, NY).

Results

The mean score of the overall perception of job satisfaction in primary healthcare staff was 3.9 (±1.01) out of one to five scale questions. This implies a high satisfaction in 71.2% (n = 215) of the sample. Nurses expressed the highest satisfaction with their roles, comprising 26.5% (n = 80) who reported high satisfaction. Second in line were physicians, demonstrating high satisfaction levels with 14.2% (n = 43). The third place was for lab technicians, 4.6% (n = 14) of whom expressed high satisfaction. Many factors were found to be significantly statistically associated with job satisfaction, including gender (p = 0.015), age (p = 0.001), job rank (p = 0.027), number of patients seen per day (p = 0.002), type of primary healthcare duty (p = 0.042), and health workers’ years of experience (p = 0.000).

Conclusion

The study revealed that HCWs at primary healthcare facilities in the Qassim region, Saudi Arabia, were highly satisfied with their jobs. Significant statistical relationships were found between job satisfaction and factors such as years of experience, type of duty in PHCCs, and number of attendees per day. To improve HCWs' job satisfaction, we recommend that they receive additional organizational support and response to feedback, a reduced workload achieved through increased staffing, and flexible work shifts. We also recommend investigating satisfaction in the private healthcare sector.

## Introduction

Raising and maintaining a good quality of life (QOL) is one of the main goals individuals work to achieve during their lifetime. QOL is defined by the World Health Organization as "an individual's perception of their position in life in the context of the culture and value systems in which they live and about their goals, expectations, standards, and concerns" [1]. A major aspect that has a significant impact on the QOL is the individual’s productivity, or in other words, career. The person’s job or career not only occupies a big part of his time but also plays a role in determining his life’s quality. Vice versa, it has been shown that having a good quality of career nature leads to an increase in productivity in addition to the positive impact it has on the person’s commitment, health, and life expectancy [2].

Job satisfaction itself has a direct impact on motivation levels and, as mentioned above, productivity. Therefore, the net result of job-satisfied individuals is higher performance in all career aspects and organizations [3]. “Job satisfaction is a measure of workers' contentedness with their jobs, whether they’re satisfied with their jobs or particular aspects/facets of the job, such as nature of work or supervision” [4]. Job satisfaction assessment tools have varied components, such as cognitive (evaluative), affective (or emotional), and behavioral [5]. To objectively estimate the job satisfaction level, validated surveys are widely used. The healthcare sector is one of the most important sectors in any country, and it has a direct impact on the level of economic advancement, growth, and civilization [6]. A major factor that interferes with healthcare entities is the disruption in the efficacy and productivity of the workforce [7]. As said respectively, job satisfaction directly interferes with the individual’s productivity. Hence, job satisfaction of healthcare workers (HCWs) is a crucial issue to discuss, investigate, and study to improve the outputs to their maximal levels eventually. A validated data collection tool known as the “Satisfaction of Employees in Health Care” (SEHC) questionnaire has proven its validity and reliability in many previous studies [8-10]. A study conducted in 2020 compared job satisfaction among different fields, showing that healthcare industry employees had the highest levels of job satisfaction, followed by education and tourism [11]. Another study that assessed job satisfaction among HCWs involving a relatively large sample showed that approximately 77% of workers were satisfied with their jobs [12]. However, a relatively large study conducted in Saudi Arabia in 2006 manifested otherwise, showing that 52.4% of physicians and 67.1% of nursing staff were dissatisfied [13]. One more recent study done in 2021 in multiple regions of Saudi supported the same results, showing that Saudi HCWs in the public sector were generally dissatisfied [14]. On the other hand, an older but significantly larger study with 626 participants conducted in the eastern region of Saudi manifested that the overall satisfaction of healthcare professionals was actually very high with a rate of 97% [15].

Given that Saudi Arabia has been developing at a faster pace, especially in the healthcare sector with the 2030 vision, and due to, as previously mentioned, the importance of job satisfaction in healthcare, with the limited studies done in the region in this regard, we planned to assess job satisfaction levels among primary HCWs, particularly in Qassim region, to find out job satisfaction levels and associated factors.

## Materials and methods

Study design and setting

A descriptive, cross-sectional, facility-based study was conducted in primary healthcare centers (PHCCs) in the Qassim region, Saudi Arabia, in a period from the 1st of October 2023 to the 10th of May 2024.

Study population

This study targeted 1020 primary healthcare providers working in 155 PHCCs in the Qassim region, including physicians, dentists, nurses, pharmacists, dental assistants, and lab technicians.

Sample size and sampling

A 95% confidence level was used to estimate a total sample size of 308 subjects from the targeted population. A 5% bound on error was applied, and a 10% non-response rate was considered. The sample size was calculated using an online sample size calculator (Open Epi) and the estimated sample size was 280. After accounting for the 10% non-response rate, the sample size was increased to 308.

By simple random sampling, 30 PHCCs were selected. From these, 308 healthcare providers were selected using a convenient non-probability sampling technique based on inclusion criteria. Inclusion criteria were all healthcare providers who were working at the time of research in PHCCs, of both genders, and those willing to participate in this study.

Data collection tool and procedures

An anonymous, pretested, self-administered, and validated digital questionnaire written in English and translated into Arabic, i.e., the "Satisfaction of Employees in Health Care" (SEHC) questionnaire, was used in this study after an extensive literature review [8-10]. The questionnaire consisted of two parts. The first part included 12 questions regarding socio-demographic characteristics, job details, and workload. The second part included the 26-item SEHC survey that identified eight domains of job satisfaction. Questions in this section were of a close-ended multiple-option format for responses using a five-point Likert scale (highly satisfied = 5, satisfied = 4, not sure = 3, dissatisfied = 2, and extremely dissatisfied = 1).

Moreover, data collection involved the use of an online questionnaire distributed via Google Forms (Google, Mountain View, CA).

Pilot study

A pilot study was conducted on 20 subjects to assess the clarity and comprehensibility of the questionnaire as well as the time needed to complete it. No modifications were necessary, so the study proceeded with the full sample size.

Statistical analysis

Data were extracted from the Excel sheet (Microsoft Corporation, Redmond, WA), entered, and analyzed using SPSS version 29 (IBM Corp., Armonk, NY). Continuous variables were expressed as mean ± standard deviation, while categorical variables were expressed as frequencies and percentages. The chi-square test was used to compare the categorical variables. P-values < 0.05 were considered statistically significant.

Scoring system of responses

The sociodemographic characteristics of the healthcare providers under investigation were analyzed using descriptive statistical methods. Job satisfaction of the staff was assessed across eight domains, with a total of 26 specific questions. These questions implemented a five-point Likert scale, with the following ratings: highly satisfied (five points), satisfied (four points), neutral (three points), dissatisfied (two points), and extremely dissatisfied (one point).

The scores for each satisfaction question were calculated separately. The value of the points for each satisfaction level was multiplied by the number of responders, and then the sum of the points was divided into four categories. Out of the total of 125 scores, ratings from 25 to 50 were classified as extremely dissatisfied, 51 to 75 as somewhat dissatisfied, 76 to 100 as somewhat satisfied, and 101 to 125 as highly satisfied. Scores <25 were classified as neutral/missing.

Question number 26 was excluded from the job satisfaction scoring as it was designed to assess the HCWs' satisfaction perception of the health facility as a whole.

Ethical considerations

A formal written approval was obtained from the Regional Research Ethics Committee, Qassim Health Cluster, Saudi Arabia (Approval No: 12291/45/607). Individual consent was also obtained upon filling out the online questionnaire. All data and info were anonymously collected, analyzed, and presented.

## Results

Participants' socio-demographic characteristics

A total of 302 out of 308 targeted participants responded to the study questionnaire, resulting in a response rate of 98.1% (n = 302). The participants included physicians representing 41.4% (n = 125), nurses representing 34.8% (n = 105), dental staff representing 13.6% (n = 41), lab technicians representing 6.6% (n = 20), pharmacists representing 2.3% (n = 7), and radiology technicians representing 1.3% (n = 4).

Table 1 presents the socio-demographic characteristics of the respondents. The female respondents accounted for 52.6% (n = 159). The average age was 35.6 (±6.99) years, ranging from 23 to 63 years. Almost half of the HCWs (50.3%, n = 152) were in the age group of 26-35 years. Most of them (51.6%, n = 156) earned between 16,000 and 37,000 SAR. Most of the participants (94.7%, n = 286) work daytime shifts in primary healthcare (PHC) facilities. The majority (84.8%, n = 256) work 40 hours per week or less. The median frequency of patients seen per day was 30, with the range being from four to 125 patients. The experience levels of HCWs vary, with the highest proportion (44.7%, n = 135) having five to 15 years of experience.

**Table 1:** Socio-demographic characteristics of the study participants, Qassim region, Saudi Arabia (2024, n = 302). N = frequency; % = percentage of frequency; ±SD = ± standard deviation; GPs = general practitioners; SAR = Saudi riyal.

Characteristics		Frequency (N)	Percentage (%)
Gender	Female	159	52.6
	Male	143	47.4
Age	<25 years	12	4
	26-35 years	152	50.3
	36-45 years	114	37.7
	46-55 years	22	7.3
	>55 years	2	0.7
	Mean (±SD)	35.6 (±6.99) years	
	Range	23-63 years	
Marital status	Unmarried	85	28.2
	Married	217	71.9
Nationality	Saudi	270	89.4
	Non-Saudi	32	10.6
Job title	Physician	125	41.4
	Nurse	105	34.8
	Dental assistant	21	6.9
	Dentist	20	6.6
	Lab technician	20	6.6
	Pharmacist	7	2.3
	Radiology technician	4	1.3
Rank	Consultants	8	2.7
	Specialists	30	9.9
	Registrars	27	8.9
	Residents	52	17.2
	GPs	45	14.9
	Other (nurses or technicians)	140	46.3
Type of primary healthcare duty	Day time	286	94.7
	On call	16	5.3
Working per week	≤40 hours	256	84.8
	>40 hours	46	15.2
Years of experience (group)	<5 years	101	33.4
	5-15 years	135	44.7
	16-26 years	54	17.9
	>26 years	12	4.0
	Mean (±SD)	9.98 (±7.86) years	
	Range	0-35 years	
Number of patients seen per day (group)	<30 patients	137	45.4
	30-49 patients	97	32.1
	50-69 patients	35	11.6
	≥70 patients	33	10.9
	Median	30 patients	
	Range	4-125 patients	
Salary per month	<5,000 SAR	5	1.7
	5,000-15,999 SAR	140	46.4
	16,000-37,000 SAR	156	51.6
	>37,000 SAR	1	0.3
Approximate distance from home to workplace	<5 km	57	18.9
	5-15 km	154	51.0
	16-26 km	44	14.6
	27-37 km	21	7.0
	>37 km	26	8.6

Assessment of HCWs’ job satisfaction

**Figure 1:**
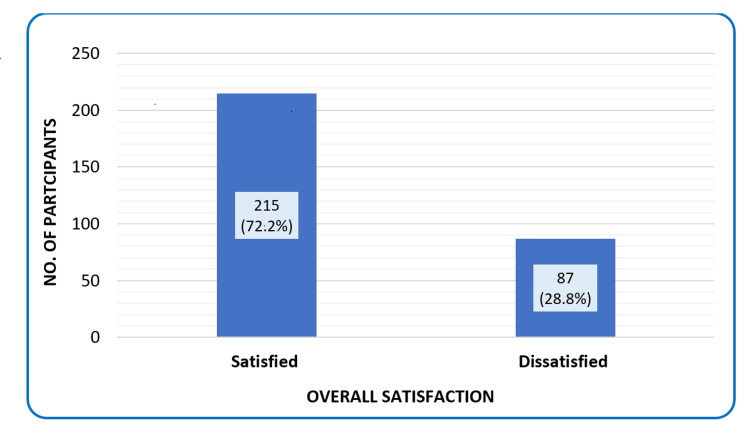
Overall healthcare workers’ satisfaction, Qassim region, Saudi Arabia (2024, n = 302).

Our study found that 72.2% (n = 215) of participants reported being satisfied overall (Figure 1).

Nurses expressed the highest satisfaction with their roles, comprising 26.5% (n = 80) who reported high satisfaction. While only 3% (n = 9) expressed extreme dissatisfaction. Second in line were physicians, also demonstrating high satisfaction levels with 14.2% (n = 43), and only 5% (n = 15) indicated extreme dissatisfaction. The third place was for lab technicians, of whom 4.6% (n = 14) expressed high satisfaction; however, none reported extreme dissatisfaction. The rest of the participants, including dentists, dental assistants, radiology technicians, and pharmacists, showed lower levels of satisfaction.

Table 2 presents the respondents' perceptions of job satisfaction across different domains. The highest satisfaction level among HCWs was reported for having accurate written job descriptions with a mean score of 4.1 (±1.29). The second highest satisfaction was reported for communication across different hierarchical levels, with a mean score of 4.0 (±1.29). Followed by satisfaction regarding support from the organization, with a mean score of 3.9 (±1.35), learning new job skills, with a mean score of 3.9 (±1.18), available chances for promotions, with a mean score of 3.9 (±1.24), and harmony with coworkers, all having the same mean score, which was 3.9 (±1.28). The lowest satisfaction of HCWs was reported for both management’s responsiveness to feedback, with a mean score of 3.4 (±1.41), and facility building, grounds, and general layout, with a mean score of 3.4 (±1.47). The rest of the different job satisfaction aspects are shown in Table 2.

**Table 2:** Healthcare workers’ job satisfaction in primary healthcare facilities, Qassim region, Saudi Arabia (2024, n = 302). N = frequency; % = percentage of frequency; mean (±SD) = mean ± standard deviation of answer’s score.

Dimension	Highly satisfied (5)	Somewhat satisfied (4)	Neutral (3)	Somewhat dissatisfied (2)	Extremely dissatisfied (1)	Mean (±SD)
	N (%)	N (%)	N (%)	N (%)	N (%)	
Supervision and administration						
1. The administration of this organization is supportive of me.	142 (47%)	60 (19.9%)	49 (16.2%)	20 (6.6%)	31 (10.3%)	3.9 (±1.35)
2. I feel encouraged by my supervisor to offer suggestions and improvements.	140 (46.4%)	47 (15.6%)	52 (17.2%)	26 (8.6%)	37 (12.3%)	3.8 (±1.42)
3. I receive the right amount of guidance and feedback from my direct supervisor.	127 (42.1%)	63 (20.9%)	58 (19.2%)	25 (8.3%)	29 (9.6%)	3.8 (±1.33)
4. I think my supervisor treats all members of the team equally.	144 (47.7%)	51 (16.9%)	42 (13.9%)	25 (8.3%)	40 (13.2%)	3.8 (±1.45)
Organization policy and support						
5. There is a clear understanding of the organization’s strategic objectives.	119 (39.4%)	56 (18.5%)	66 (21.9%)	27 (8.9%)	34 (11.3%)	3.7 (±1.37)
6. I feel I can easily communicate with members from all levels of this organization.	152 (50.3%)	61 (20.2%)	42 (13.9%)	24 (7.9%)	23 (7.6%)	4 (±1.29)
7. If something unusual comes up, I know who to go to for a solution.	146 (48.3%)	51 (16.9%)	53 (17.5%)	22 (7.3%)	30 (9.9%)	3.9 (±1.35)
8. The management makes changes based on my suggestions and feedback.	96 (31.8%)	53 (17.5%)	76 (25.2%)	32 (10.6%)	45 (14.9%)	3.4 (±1.41)
9. My work is evaluated based on a fair system of performance standards.	140 (46.4%)	62 (20.5%)	48 (15.9%)	23 (7.6%)	29 (9.6%)	3.7 (±1.33)
10. I have an accurate written job description.	167 (55.3%)	57 (18.9%)	29 (9.6%)	27 (8.9%)	22 (7.3%)	4.1 (±1.29)
11. The organization's rules make it easy for me to do a good job.	120 (39.7%)	67 (22.2%)	55 (18.2%)	28 (9.3%)	32 (10.6%)	3.7 (±1.35)
12. My department provides all the equipment, supplies, and resources necessary for me to perform my duties.	100 (33.1%)	54 (17.9%)	46 (15.2%)	50 (16.6%)	52 (17.2%)	3.3 (±1.50)
13. This facility's buildings, grounds, and layout are adequate for me to perform my duties.	101 (33.4%)	57 (18.9%)	54 (17.9%)	42 (13.9%)	48 (15.9%)	3.4 (±1.47)
Advancement						
14. I am provided with all the training necessary for me to perform my job.	115 (38.1%)	67 (22.2%)	49 (16.2%)	42 (13.9%)	29 (9.6%)	3.7 (±1.36)
15. I have learned many new job skills in this position.	136 (45%)	73 (24.2%)	52 (17.2%)	27 (8.9%)	14 (4.6%)	3.9 (±1.18)
16. I believe that there is an opportunity for individual career growth and development within the organization.	108 (35.8%)	69 (22.8%)	56 (18.5%)	36 (11.9%)	33 (10.9%)	3.6 (±1.36)
Promotion						
17. I am appropriately recognized when I perform well at my regular work duties.	120 (39.7%)	60 (19.9%)	52 (17.2%)	33 (10.9%)	37 (12.3%)	3.6 (±1.41)
18. I am satisfied with my chances for promotion.	144 (47.7%)	69 (22.8%)	45 (14.9%)	24 (7.9%)	20 (6.6%)	3.9 (±1.24)
Job content						
19. The amount of work I am expected to do each week is reasonable.	113 (37.4%)	71 (23.5%)	52 (17.2%)	33 (10.9%)	33 (10.9%)	3.7 (±1.36)
Pay						
20. I think I’m fairly paid for what I do/I’m satisfied with my salary.	121 (40.1%)	65 (21.5%)	62 (20.5%)	32 (10.6%)	22 (7.3%)	3.8 (±1.28)
Co-workers						
21. My team is an inspiration for me to do my best at the job.	137 (45.4%%)	55 (18.2%)	50 (16.6%)	31 (10.3%)	29 (9.6%)	3.8 (±1.36)
22. My coworkers and I work well together.	145 (48%)	65 (21.5%)	48 (15.9%)	19 (6.3%)	25 (8.3%)	3.9 (±1.28)
Overall, job						
23. My environment at work helps me strike the right work-life balance.	116 (38.4%)	61 (20.2%)	59 (19.5%)	31 (10.3%)	35 (11.6%)	3.6 (±1.38)
24. I think I will be working for the same organization in the next 2 years.	119 (39.4%)	56 (18.5%)	42 (13.9%)	37 (12.3%)	48 (15.9%)	3.5 (±1.50)
25. I would recommend this health facility to other workers as a good place to work.	125 (41.4%)	58 (19.2%)	41 (13.6%)	35 (11.6%)	43 (14.2%)	3.6 (±1.47)
26. How would you rate this health facility as a place to work?	118(39.1%)	82(27.2%)	40(13.2%)	30(9.9%)	32(10.6%)	3.7 (±1.35)

Moreover, our study revealed several factors significantly associated with job satisfaction.

Table 3 illustrates the relationship between the socio-demographic characteristics of HCWs and their overall job satisfaction. Gender was found to significantly relate to job satisfaction (p = 0.015), with 25.8% of males (n = 78) reporting high satisfaction. Age also demonstrated a significant relationship with job satisfaction (p = 0.001). HCWs aged 26-35 years comprised the highest number of highly satisfied responses (18.5%, n = 56). A notable significant association with job satisfaction was found among different ranks (p = 0.027). Years of experience also yielded highly significant results (p = 0.000), with health workers having five to 15 years of experience showing the highest number of highly satisfied responses of 23.2% (n = 70). Similarly, the average number of patients seen per day also had a strong role in job satisfaction (p = 0.002). Those who were seeing fewer than 30 patients reported higher satisfaction levels compared to those seeing more patients. Professionals engaged in daytime PHC shifts reported 44.7% (n = 135) high satisfaction. Conversely, on-call PHC duty professionals exhibited lower satisfaction, with only 2.3% (n = 7) reporting high satisfaction. This also implies a statistically significant relationship (p = 0.042).

**Table 3:** The association between healthcare workers’ characteristics and the level of job satisfaction in Qassim region, Saudi Arabia (2024, n = 302). N = frequency; % = percentage of frequency; GP= general practitioner; SAR = Saudi riyal. * Statistically significant (p-value < 0.05).

Variables	Highly satisfied, N (%)	Somewhat satisfied, N (%)	Somewhat dissatisfied, N (%)	Extremely dissatisfied, N (%)	p-value
Gender					0.015*
Female	64 (21.2%)	39 (12.9%)	36 (11.9%)	20 (6.6%)	
Male	78 (25.8%)	34 (11.3%)	25 (8.3%)	6 (2%)	
Age (years)					0.001*
<25	2 (0.7%)	1 (0.3%)	7 (2.3%)	2 (0.7%)	
26-35	56 (18.5%)	43 (14.2%)	34 (11.3%)	19 (6.3%)	
36-45	68 (22.5%)	24 (7.9%)	17 (5.6%)	5 (1.7%)	
46-55	15 (5%)	4 (1.3%)	3 (1%)	0 (0%)	
>55	1 (0.3%)	1 (0.3%)	0 (0%)	0 (0%)	
Marital status					0.109
Single	31 (10.3%)	25 (8.3%)	22 (7.3%)	7 (2.3%)	
Married	111 (36.8%)	48 (15.9%)	39 (12.9%)	19 (6.3%)	
Nationality					0.20
Saudi	123 (40.7%)	65 (21.5%)	59 (19.5%)	23 (7.6%)	
Non-Saudi	19 (6.3%)	8 (2.6%)	2 (0.7%)	3 (1%)	
Job title					0.23
Dental assistant	10 (3.3%)	5 (1.7%)	3 (1%)	3 (1%)	
Dentist	9 (3%)	5 (1.7%)	5 (1.7%)	1 (0.3%)	
Lab technician	14 (4.6%)	3 (1%)	3 (1%)	0 (0%)	
Nurse	60 (19.9%)	21 (7%)	17 (5.6%)	7 (2.3%)	
Pharmacist	3 (1%)	2 (0.7%)	2 (0.7%)	0 (0%)	
Physician	43 (14.2%)	36 (11.9%)	31 (10.3%)	15 (5%)	
Radiology technician	3 (1%)	1 (0.3%)	0 (0%)	0 (0%)	
Rank					0.027*
GP	22 (7.3%)	10 (3.3%)	11 (3.6%)	2 (0.7%)	
Resident	15 (5%)	15 (5%)	17 (5.6%)	5 (1.7%)	
Registrar	8 (2.6%)	8 (2.6%)	5 (1.7%)	6 (2%)	
Specialist	14 (4.6%)	10 (3.3%)	3 (1%)	3 (1%)	
Consultant	3 (1%)	3 (1%)	1 (0.3%)	1 (0.3%)	
Other (nurses and technicians)	80 (26.5%)	27 (8.9%)	24 (7.9%)	9 (3%)	
Years of experience					
<5 years	30 (9.9%)	30 (9.9%)	32 (10.6%)	9 (3%)	
5-15 years	70 (23.2%)	24 (7.9%)	25 (8.3%)	16 (5.3%)	0.000*
16-26 years	34 (11.3%)	15 (5%)	4 (1.3%)	1 (0.3%)	
>26 years	8 (2.6%)	4 (1.3%)	0 (0%)	0 (0%)	
Type of primary healthcare duty					0.042*
Daytime	135 (44.7%)	65 (21.5%)	61 (20.2%)	25 (8.3%)	
On-call	7 (2.3%)	8 (2.6%)	0 (0%)	1 (0.3%)	
Working hours/week					0.382
Less than 40	120 (39.7%)	65 (21.5%)	48 (15.9%)	23 (7.6%)	
More than 40	22 (7.3%)	8 (2.6%)	13 (4.3%)	3 (1%)	
Average patients seen per day					
<30 patients	58 (19.2%)	37 (12.3%)	30 (9.9%)	12 (4%)	0.002*
30-49 patients	36 (11.9%)	23 (7.6%)	27 (8.9%)	11 (3.6%)	
50-69 patients	27 (8.9%)	7 (2.3%)	1 (0.3%)	0 (0%)	
>70 patients	21 (7%)	6 (2%)	3 (1%)	3 (1%)	
Salary per month					0.74
<5,000 SAR	3 (1%)	1 (0.3%)	1 (0.3%)	0 (0%)	
5,000-15,999 SAR	75 (24.8%)	29 (9.6%)	24 (7.9%)	12 (4%)	
16,000-26,999 SAR	58 (19.2%)	37 (12.3%)	31 (10.3%)	12 (4%)	
27,000-37,000 SAR	5 (1.7%)	6 (2%)	5 (1.7%)	2 (0.7%)	
>37,000 SAR	1 (0.3%)	0 (0%)	0 (0%)	0 (0%)	
Approx. distance from home to workplace					0.601
<5 km	31 (10.3%)	11 (3.6%)	11 (3.6%)	4 (1.3%)	
5-15 km	77 (25.5%)	39 (12.9%)	28 (9.3%)	10 (3.3%)	
16-26 km	17 (5.6%)	11 (3.6%)	10 (3.3%)	6 (2%)	
27-37 km	10 (3.3%)	5 (1.7%)	4 (1.3%)	2 (0.7%)	
>37 km	7 (2.3%)	7 (2.3%)	8 (2.6%)	4 (1.3%)	

## Discussion

As previously emphasized, job satisfaction plays a significant role in determining the quality and efficiency of outputs, especially in the healthcare sector [7]. Our study revealed the average job satisfaction score of primary HCWs in Qassim PHCCs, which was 3.9 on a scale of one to five. This implies a high satisfaction among the sample. This was in line with other studies done globally targeting job satisfaction among different work industries, including insurance, finance, tourism, outsourcing, education, and obviously, as mentioned, health [11]. Studies done internationally on the healthcare sector [12,16] and studies done in Saudi [15] revealed that even though participants reported being stressed, they showed relatively high job satisfaction levels. On the other hand, a study done in Qatar with a relatively large sample of 2067 subjects showed that 41.8% of the sample were dissatisfied with their jobs [17].

Among our sample, nurses showed the highest levels of satisfaction, with 26.5% (n = 80) reporting top satisfaction levels. In spite of that, a study done on 100 staff nurses in India revealed that about 92% of them show ambivalent levels of satisfaction [18].

Our study also investigated the relationship between workload (by the number of patients seen per day) and job satisfaction, which gave us a highly significant correlation (p = 0.002). Other studies have also come with similar results globally [19,20], and in more local Arabic countries as well (Egypt) [21]. Age was also found as one of the significant factors associated with job satisfaction in our study. Similarly, a study in Jordan [22] done in 2021 and Egypt [21] done in the same year supported this finding.

Interestingly, even though salary/income was perceived positively with a high level of satisfaction in this study, many previous studies manifested otherwise. A study done in China revealed that the highest levels of dissatisfaction were actually among items related to monetary factors [16]. Other studies also represented similar results. A study done in Italy [23] with more than 7,000 subjects revealed that having a higher salary was positively correlated with satisfaction, and another recent study done on PHCCs in Greece [24] showed that subjects were dissatisfied with their salaries.

Our data collection tool, i.e., the Satisfaction of Employees in Health Care (SEHC) survey, has been adopted by many previous studies [9,10]. Another point in favor of the tool is that a study found that it has high reliability (Cronbach's alpha = 0.942) and validity (r = 0.77 and 0.76, both p < 0.05) [8]. This, in turn, supports the accuracy and reliability of our results.

Limitations

On the other hand, having a relatively low number of respondents in on-call duty, high age group, high rank, and experience gives us a less accurate interpretation. An additional limitation that should be acknowledged is the cross-sectional design, which limits our ability to obtain the causal effects of job satisfaction. Moreover, the reliance on self-reported data may potentially influence the accuracy of responses. Furthermore, the generalizability of our findings may be limited to the Qassim region of Saudi Arabia and may not reflect the experiences of primary HCWs in other regions or countries. Lastly, we have not collected data from private sittings. As probably known, there is a core difference regarding workload, qualifications of employees, and experience between the public and private sectors. Supporting this, several studies proved a difference in this regard between the two sectors [25,26].

## Conclusions

In our study, we found that primary HCWs in the Qassim region of Saudi Arabia reported high job satisfaction. Nurses, in particular, reported the highest levels of satisfaction compared to other primary HCWs. We identified several factors that showed a significant statistical association with job satisfaction levels, including gender (p = 0.015), age (p = 0.001), rank (p = 0.027), years of experience (p = 0.000), type of primary healthcare duty (p = 0.042), and the number of patients seen per day (p = 0.002).

To improve HCWs' job satisfaction, it is recommended that they receive additional organizational support and response to feedback, a reduced workload achieved through increased staffing, and flexible work shifts. Additionally, for a better wholesome assessment of satisfaction in health care, we recommend conducting studies in the private sector as well.
